# Surface Characterization of Electro-Assisted Titanium Implants: A Multi-Technique Approach

**DOI:** 10.3390/ma13030705

**Published:** 2020-02-05

**Authors:** Stefania Cometa, Maria A. Bonifacio, Ana M. Ferreira, Piergiorgio Gentile, Elvira De Giglio

**Affiliations:** 1 Jaber Innovation s.r.l., 00144 Rome, Italy; stefania.cometa@jaber.it; 2 Department of Chemistry, University of Bari “Aldo Moro”, 70126 Bari, Italy; maria.bonifacio@uniba.it; 3 School of Engineering, Newcastle University, Newcastle NE1 7RU, UK; Ana.Ferreira-Duarte@newcastle.ac.uk (A.M.F.); piergiorgio.gentile@newcastle.ac.uk (P.G.)

**Keywords:** analytical characterization, mechanical tests, titanium, polymeric coatings, surface properties, physico-chemical study, morphology

## Abstract

The understanding of chemical–physical, morphological, and mechanical properties of polymer coatings is a crucial preliminary step for further biological evaluation of the processes occurring on the coatings’ surface. Several studies have demonstrated how surface properties play a key role in the interactions between biomolecules (e.g., proteins, cells, extracellular matrix, and biological fluids) and titanium, such as chemical composition (investigated by means of XPS, TOF-SIMS, and ATR-FTIR), morphology (SEM–EDX), roughness (AFM), thickness (Ellipsometry), wettability (CA), solution–surface interactions (QCM-D), and mechanical features (hardness, elastic modulus, adhesion, and fatigue strength). In this review, we report an overview of the main analytical and mechanical methods commonly used to characterize polymer-based coatings deposited on titanium implants by electro-assisted techniques. A description of the relevance and shortcomings of each technique is described, in order to provide suitable information for the design and characterization of advanced coatings or for the optimization of the existing ones.

## 1. Introduction

In orthopedic and dental applications, mechanical strength and biocompatibility are crucial properties for a good implant performance, and polymeric coatings play a pivotal role in improving the biological response [1]. By carefully selecting the appropriate polymer composition, the properties of the final composite can be tuned. Firstly, coatings can be exploited to extend implant lifespan by creating an anti-wear barrier, which prevents corrosion and stress-shielding phenomena [2]. Secondly, a thin layer of polymer to cover a titanium implant may enhance the integration of the surrounding tissue, improving the surface bioactivity [3]. Moreover, one of the major goals of a titanium coating lies in the prevention of infections, which are a frequent cause of implant failure. Indeed, bare titanium is easily colonized by bacteria, resulting in biofilm growth, corrosion pits formation, and subsequent deterioration of mechanical performance [4].

Several techniques have been developed to synthesize or deposit polymeric and/or hydrogel coatings, including electro-assisted strategies [5]. In particular, electrophoresis, electrochemical polymerization (or electrosynthesis), electrospark deposition, electrospraying, and electrospinning represent the main examples of electricity-aided metal surface modification. For all these techniques, even if with quite different experimental setups, electrical phenomena are always involved.

These strategies, here summarized in the expression “electro-assisted titanium implants”, are employed to achieve homogeneous, thin polymer coatings. Moreover, these procedures may not need to apply harsh conditions (e.g., high temperature, high pressure, toxic reagents), and green solvents can be used. In addition, the electro-assisted techniques allow the deposition of more than one material on the conductive substrate, leading to complex, multifunctional surfaces. In addition, different nano-structures, such as nano-fibers, nano-rods, nano-wires, nano-tubes, nano-sheets, nano-particles, nano-sponges, or nano-composites can be engineered.

Overall, polymeric coatings represent a useful strategy to tune the surface features of titanium implants, controlling the interactions with the host tissue, while preserving the bulk key properties [6]. In this respect, polymers provide cells with a plethora of chemical, topographical, and mechanical cues [7]. Indeed, the polymers’ physico-chemical features (e.g., surface chemistry, wettability, roughness, topography, and stiffness) affect cell adhesion, morphology, and proliferation [8]. Furthermore, the three-dimensional architecture of polymeric materials, including porosity, impact cell motility, as well as cell metabolism and transport processes [9]. Recent findings highlight that polymer orientation, alignment, and polarization drive cell differentiation, triggering cytoskeletal rearrangements and focal adhesion structuring, which induce specific intracellular signaling pathways [10,11]. Hence, polymeric coatings represent a promising approach to address the proper colonization of an implanted material. In addition, cell adhesion phenomena have to face bacterial competition on the implant. In this respect, the biomaterial physico-chemical properties play a crucial role, as reported by do Nascimento et al., who described two different microbial populations on titanium and zirconia dental implants [12]. Indeed, a higher number of pathogenic bacteria were detected on titanium implants as compared to zirconia, highlighting how the composition and topography affect bacterial colonization and determine the growth of a selected, biofilm-forming microbiota.

In this scenario, major knowledge of the potentialities of analytical techniques devoted to investigating post-deposition properties of the coatings enables scientists to carry out more reproducible and controllable electro-deposited polymer films on titanium, with predictable interactions with living tissues. We believe that a comprehensive characterization of electro-assisted polymers at various structural levels might improve the design of such materials, which could be finally scaled up on an industrial level.

The chemical, physical, morphological, and mechanical properties of polymeric coatings drive many of their measurable features (e.g., porosity, wettability, resistance to mechanical stress). Thus, classical and advanced characterization techniques are essential to expand the current knowledge on the surface properties of coated titanium orthopedic and orthodontic implants.

One technique alone could not afford all the desired comprehension of the surface properties of thin films, especially when the studied systems are complex, such as the electro-assisted ones. A multi-technique approach, providing complementary information, is often mandatory. This review aims to summarize the main, commonly employed techniques (shown in Table 1) to study polymeric coatings, deposited on titanium by electro-assisted routes, from an analytical and mechanical point of view. As such, the review provides a synthetic, updated outline for senior scientists skilled in biomaterials research but, at the same time, it could be useful as a basic manual for first readers approaching the characterization of polymeric coatings.

## 2. Surface Chemical Composition

### 2.1. X-ray Photoelectron Spectroscopy

X-ray photoelectron spectroscopy (XPS), also called electron spectroscopy for chemical analysis (ESCA), is one of the key analytical techniques widely used to characterize the surface chemistry of solid materials and, in particular, polymer-based biomaterials and coatings. XPS is a highly surface-sensitive technique because only the electrons emitted from the outermost surface (of about 10 nm) can escape without energy loss.

XPS is typically performed by acquiring a survey scan at low resolution, then scanned at high resolution on smaller eV windows (typically of 20 eV or less). Survey scans, which cover all the eV range, are used to identify and quantify major elements present on the materials as atomic percentage. Semi-quantitative information can be obtained by measuring the relative peak areas of specific elemental lines and by applying appropriate atomic relative sensitivity factors (RSF), empirically derived from databases or analysis of standard samples.

By high-resolution spectra more specific information on the so-called chemical shift can be obtained. This slight binding energy (BE) variation is related either to the oxidation state, to the nature of chemical bonds, or to the presence of different neighboring atoms [13]. For example, in polymers, the acquisition of the carbon region, such as the C1s signal, makes it possible to obtain qualitative and semi-quantitative information of the chemical bonds involving carbon atoms on the surface, learning the exact chemical structure of the polymer surface layers. In addition, surface chemical information from inorganic biomaterials or inorganic coatings can be also obtained. For example, researchers often study the surface chemical properties of commercially pure titanium (cp-Ti) and Ti alloys by means of XPS [14,15,16]. In some cases, the study of high-resolution Ti2p spectra of differently-treated Ti surfaces added some relevant information hardly obtainable by means of other techniques [17].

As far as electrodeposited polymer coatings on titanium are concerned, a remarkable study reported on the employment of XPS as a primary characterization technique [18]. In particular, one of the most significant works carried out in about three decades concerned the strategic role of XPS in the characterization of electrosynthesized thin films, for the development of biosensors, biocompatible coatings, and active layers for gas sensors [19]. Related studies have developed new electrosynthesized polymer coatings on Ti or Ti alloys, with different functions. These polymeric coatings were mainly poly-pyrrole [20,21,22] or poly-acrylate based [23,24,25]. XPS was exploited as the main chemical characterization technique to obtain information both on polymer coating stoichiometry and the homogeneity of titanium coverage. Moreover, XPS analysis can supply much other evidence. For example, the presence of an organophosphoric monomer in the P(HEMA-MOEP) copolymer coating, electrosynthesized on titanium, was clearly highlighted by the presence of P2p signal in the survey spectrum of this system [26]. Furthermore, ciprofloxacin component on the surface of polyacrylate-based hydrogels electropolymerized on titanium was detected and quantified by XPS due to the presence of fluorine in the molecule, which is a typical marker element for XPS investigation [27]. Again, elements such as silver [28,29] or gallium [30], employed for the development of antibacterial polyacrylate electrosynthesized coatings on titanium, have been easily detected by XPS measurements.

**Figure 1:**
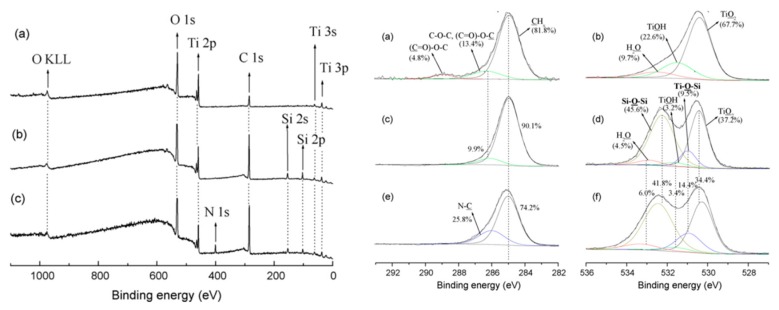
**On the left**: X-ray photoelectron spectroscopy (XPS) survey spectra of (**a**) titanium substrates and titanium substrates modified (2 h of reaction) by (**b**) HTCS and (**c**) PyHTCS. **On the right**: XPS of C1s and O1s high resolution spectra of titanium substrates (**a**,**b**) and titanium substrates modified (2 h of reaction) by HTCS (**c**,**d**) PyHTCS (**e**,**f**). (Reprinted with permission from [35], Elsevier 2008 copyright n. 4710120673412).

Furthermore, scientists in the field have exploited this technique to characterize electrodeposited coatings on titanium [31,32,33,34]. Mekhalif and coworkers reported the silanization of titanium substrates and subsequent electropolymerization of pyrrole on the resulting silanized substrate. In order to assess the stoichiometry of the grafted layers, C/Si and Si/N ratios were obtained by an XPS survey spectra, whilst a detailed curve fitting of C1s evidenced the presence of a new peak related to the C–N bond typical of pyrrole moieties (Figure 1). An accurate study of the O1s signal proved the formation of Ti–O–Si and Si–O–Si new linkages [35]. Following this work, the same research group proposed two different acidic conditions to graft 4-pyrrolyphenyldiazonium (Py-PD) on NiTi by studying electropolymerization of pyrrole on modified surfaces with Py-PD as adhesion promoter. This elegant modification procedure was accurately investigated by analyzing C1s, N1s, and Ti2p signals of the bare NiTi and Py-PD grafted films [36].

Erakovic et al. employed the electrophoretic deposition (EPD, i.e., deposition of a polymer present in the electrolyte solution, by attraction to the metal electrode) to obtain hydroxyapatite/lignin composite coatings on titanium substrates. Moreover, the coatings were successfully sintered at 900 °C. First, XPS analysis revealed differences between sintered and non-sintered hydroxyapatite surfaces with and without lignin. Moreover, the Ca:P ratio obtained for the non-sintered hydroxyapatite and for hydroxyapatite/lignin coatings was ascribable to synthetic apatite phases. After sintering, the Ca:P ratio significantly increased and the authors hypothesized a diffusion of phosphorous ions into the Ti surface, with the formation of CaO as a result of HA decomposition, especially in samples without lignin [32]. The same authors used silver-doped hydroxyapatite to obtain an antimicrobial composite coating with lignin on titanium via EPD. Curve fitting of Ca2p, P2p (indicating the presence of hydroxyapatite), C1s, and O1s (suggesting the presence of the polymer lignin) high-resolution signals was performed. Furthermore, no silver peak was detected because of its very small superficial amount [37].

Sirivisoot and coworkers firstly obtained multi-walled carbon nanotubes grown out of anodized nanotubular Ti (MWNT–Ti) using chemical vapor deposition. Successively, MWNT–Ti and commercially-pure Ti were used as templates to electrosynthesize PPy by cyclic voltammetry (i.e., an electrochemical technique where the working electrode potential was cycled, and the result was the electrochemical oxidation or reduction of the monomer or the electrochemical initiator in an electropolymerization setup) and, finally, a conjugation with PLGA moieties was achieved, entrapping dexamethasone (Dex) in the structure, to obtain PLGA–PPy[Dex]–Ti samples. XPS analysis revealed the presence of Dex in the coating through the C–F bond described in the curve fitting of C1s. Moreover, the N1s curve fitting indicated the presence of three contributions. First, deprotonated pyrrole nitrogen (imine-like) –N=; second, neutral pyrrole nitrogen (amine-like) –NH–; third, positive pyrrole nitrogen –N^+^–. The relative ratio of the imine-like (–N=) to amine-like (–NH–) components was helpful to measure the intrinsic oxidation state of PPy [38].

Wang et al. carried out electrodeposition on titanium of an alginate/chitosan layer-by-layer composite coating. XPS analysis allowed the quantification of the extent of protonation of amine groups, through the examination of the N1s high-resolution spectra, indicating that less than half of the chitosan amine groups were protonated, thus chitosan and alginate films could electrostatically interact and form chitosan/alginate layer-by-layer composite coatings on titanium substrates [39].

Yan et al. employed graphene oxide cross-linked gelatin as reinforcement fillers in hydroxyapatite coatings by the electrochemical deposition process on TiO_2_ nanotube arrays (TNs), grown on titanium by electrochemical anodization. The XPS technique was applied to determine the quantitative surface composition of coatings. Moreover, the C1s curve fitting of the composite coating, in particular, revealed the appearance of N–C=O groups, indicative of the successful condensation reaction (amidation) between amino groups and carboxyl groups [40].

GhavamiNejad and co-workers carried out dopamine electropolymerization on titanium substrates, comparing the results with the classical poly(dopamine) dip coating. Since poly(dopamine) can be used as a reducing agent for metal ions into nanoparticles, in this article in situ Ag nanoparticle formation was also evaluated. The XPS results for both samples showed the presence of Ag3d and Ag3p at BE values, indicating the successful reduction of silver metal ion to the silver nanoparticles at zero-valent state. Moreover, XPS highlighted that the content of silver nanoparticles in the electrosynthesized poly(dopamine) sample was higher than in the dip-coated one [41].

Dosic et al. developed a three-component composite coating, based on graphene, chitosan, and hydroxyapatite, by cathodic EDP on Ti substrates. XPS results confirmed the presence of graphene, since the C1s peak at BE ~285 eV was ascribed to aromatic hydrocarbons, consistent with the graphene honeycomb structure [42].

Moreover, Simi et al. fabricated titania nanotube arrays on a Ti substrate by the potentiostatic anodization process. Successively, the deposition of polypyrrole into a titania nanotube structure was achieved by normal pulse voltammetry electrodeposition. This electrochemically assembled polypyrrole/titania nanotube array (PPy/TNTA) surface was characterized by different techniques, including XPS. The PPy/TNTA surface was composed by Ti2p, O1s, N1s, and C1s. In addition, both C1s and N1s signals were deconvoluted by contributions typical of neutral PPy, while both Ti2p and O1s curve fittings revealed the presence of Ti–O bonds [43].

Finally, Stevanovic et al. carried out a composite coating based on chitosan hydroxyapatite and gentamicin by EPD on pure titanium plates. XPS, with FTIR analysis, was used to confirm that the formation of composite coatings on titanium was due to intermolecular hydrogen bonds [44].

Overall, XPS is undeniably a fundamental technique for electro-assisted titanium implants. Other information obtainable is, for example, the partial or total covering of the substrate and an idea of the coating thickness, indirectly gained by acquiring XPS spectra over different sampling points. Indeed, if the coatings are too thin, the titanium signal, relevant to the substrate, could emerge. In summary, although XPS is not a new technique, it is expected to be a workhorse in future biomaterials research, especially to study organic biomaterials such as polymer coatings.

### 2.2. Time-of-Flight Secondary Ion Mass Spectrometry

Time-of-Flight Secondary Ion Mass Spectrometry (TOF-SIMS) provides elemental, chemical state, and molecular information from the surfaces of solid materials. This technique allows the detection most of the elements in the periodic table, including hydrogen and helium (differently from XPS), as well as atomic ions and molecular ions at low concentrations. The average depth of analysis is approximately 1 nm, with an ultimate spatial resolution of less than 0.1 µm. Information on spatial distribution can be obtained by scanning a micro-focused ion beam across the sample surface, obtaining a 2D imaging of the surface. A 3D reconstruction of the TOF-SIMS data is also possible, since the surface orientation and topography of the sample could affect the trajectory of the primary ion beam. Indeed, sputter yields not only depend on the primary ion energy but also on the incidence angle, which, in turn, changes depending on the surface topography. Depth distribution information is achieved by combining TOF-SIMS measurements with ion sputtering (giving rise to a 3D analysis) [45]. The sensitivity of the SIMS technique depends on the yield of secondary ion sputtering, which, in turn, is a function of different factors, such as type, energy, and incidence angle of the primary ion beam, as well as the matrix effect. Therefore, an accurate quantification of SIMS results in terms of surface composition is often limited. On the other hand, the sensitivity of the SIMS technique is very high, and the measurement of trace elements at ppb-level is possible [46]. In the orthopedic and dental biomaterials fields, TOF-SIMS is gaining an emerging role for titanium implants characterization.

**Figure 2:**
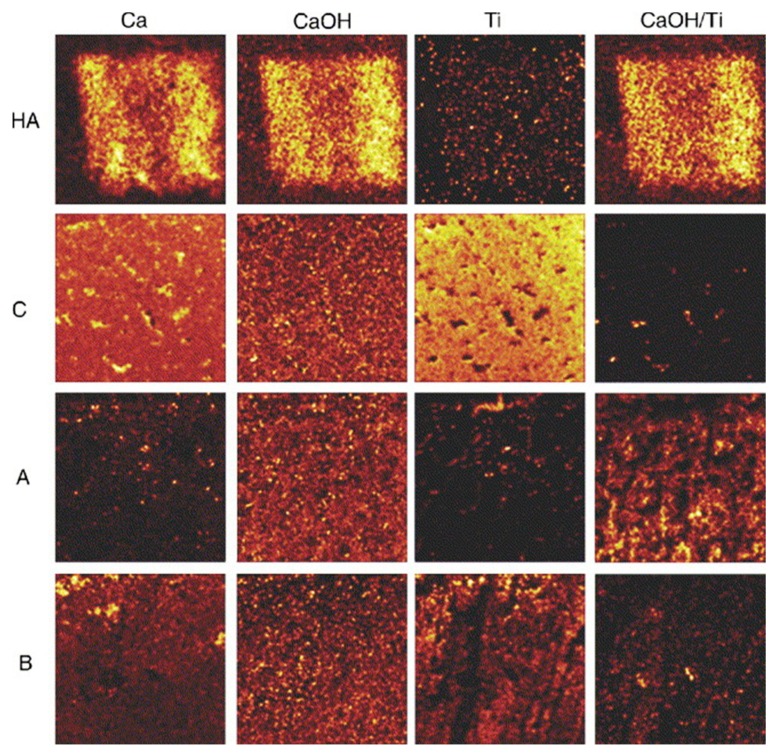
Time-of-Flight Secondary Ion Mass Spectrometry (TOF-SIMS) images showing the secondary ion distributions for the hydroxyapatite control (HA), not anodized titanium control after implantation (C), and the implanted porous surfaces A and B. The figure shows the results for Ca^2+^, CaOH, Ti, and a normalization image calculated from CaOH and Ti images of the sample. All images were normalized to the total ion count. The field of view is 200 μm × 200 μm, except for HA, which is 500 μm × 500 μm. (Reprinted with permission from [47], Elsevier 2006 copyright n. 4710120857878).

For example, Eriksson et al. investigated mineralization (hydroxyapatite formation) on different titanium surfaces implanted in bone after one week using TOF-SIMS (Figure 2) [47]. Moreover, Metoki et al. studied the electrodeposition of calcium phosphate (CaP) on a titanium alloy, covered with self-assembled monolayers (SAMs). The authors focused on the influence of chain length, end-group charge, and anchoring group [48]. In this respect, TOF-SIMS was applied to obtain information about the early phase content of the coating, indicating that Ca-rich phases were formed initially in the presence of SAMs. TOF-SIMS has been often used in combination with XPS to fully characterize the electrosynthesized polymers. In this respect, Idla et al. carried out a pioneering work, in which SIMS, together with XPS, was employed for the characterization of electrosynthesized PPy films on titanium substrates. SIMS made it possible to perform a depth profile analysis of the films and to investigate the polymer/electrode interface [49].

Moreover, De Giglio et al. provided indirect information on the electroactivity of electrosynthesized polymers by TOF-SIMS, thanks to the amount of electrolyte halogen-containing anions entrapped into polypyrrole-3-carboxylic acid (PPy-3-carbox) films [20]. The presence of halogen-containing species at the surface was confirmed in a successive work, with other important features highlighted by SIMS, such as the presence of silver traces in the electrolytic solution and of oxidized species, likely containing the carboxylic groups of PPy-3-carbox films. TOF-SIMS investigations on 3-pyrrole-acetic electrosynthesied coatings underlined a decarboxylation of the rings and a decrease in unsaturation degree [21].

Finally, Teo and co-workers electrosynthesized on titanium both oxidized and reduced films of polypyrrole-poly(vinylsulfonic acid, sodium salt) (PPy-PVS). TOF-SIMS analysis evidenced that PVS anions were more present on the electrolyte side surface than the electrode side surface, giving a rough surface of reduced PPy-PVS film, whereas more PPy chains were observed on the surface of oxidized PPy-PVS film [50].

Surely, TOF-SIMS and XPS analyses are complementary and the combination of these two techniques is essential to fully understand the physico-chemical composition of polymer surfaces. Indeed, the limitations present in one technique could be easily overcome by the other. For example, XPS cannot distinguish isotopes, while TOF-SIMS is able to. The XPS detection limit is 0.1%, while TOF-SIMS reaches the ppb order. Elemental or molecular imaging is possible only for TOF-SIMS. On the other hand, quantification is difficult with TOF-SIMS, while it can be routinely performed by XPS. The ongoing developments of TOF-SIMS, such as employment of multivariate analysis methods for data mining, or the use of cluster-ion beams and metal-assisted SIMS, supplied significant improvements and new application areas to this technique.

### 2.3. Attenuated Total Reflectance Fourier-Transform Infrared Spectroscopy

Fourier-transform infrared (FTIR) analysis is a basic and routinely used polymeric biomaterials characterization technique, extensively employed to identify polymeric materials, providing useful qualitative information in a short time. By matching the FTIR spectrum of an unknown material with that of a known material (indeed, reference spectra for common polymers are commercially available, or may be acquired), the fingerprinting of the material can be carried out.

There are different acquisition modes of FTIR spectroscopy. The standard mode is transmission IR spectroscopy, which involves the passage of an infrared beam through the specimen. In this case, at least partial transmission of the infrared beam through the sample is required. Therefore, the accumulated spectrum measures the bulk, in place of the surface’s properties. For this reason, the transmission mode is not useful for surface analysis of biomaterials or coatings. Alternatively, reflecting modes, such as attenuated total reflectance (ATR) FTIR spectroscopy, are commonly exploited for surface characterization of biomaterials. It is worth noting that the XPS technique is much more surface-sensitive than ATR-FTIR spectroscopy, in which the infrared beam can penetrate from several hundred nanometers to more than 1 µm. One of the major disadvantages of this technique is that intimate contact between the coating and the ATR crystal is not so simple to initiate, especially when films are rough and the underlying titanium substrate is hard and not perfectly flat. On the other hand, sample inhomogeneity features can be recently minimized by calculating second derivative spectra.

As far as coating synthesis is concerned, ATR-FTIR spectroscopy is widely used to confirm coating formation on titanium [51,52,53,54,55,56,57,58]. For example, the hydroxyapatite/lignin composite coatings on titanium implants, developed by Erakovic et al., were also studied by means of ATR-FTIR. This analysis underlined the absence of carbonate peaks, thus no formation of calcium carbonate or calcium oxide in the apatite occurred. Hence, the reaction was completed and the pure phase HA was obtained [32].

The same authors used silver-doped hydroxyapatite to develop an antimicrobial, lignin-based, composite coating on titanium via EPD. ATR-FTIR analysis was employed to investigate the coating surface after seven days of soaking in simulated body fluid (SBF). The broad absorbance band due to OH-stretching, with higher intensity after immersion, as well as the presence of three peaks ascribable to the vibrational band of carbonate groups, revealed the formation of a new bone-like apatite layer on the coating surface [37].

Furthermore, Ungureanu et al. electrodeposited composites based on polypyrrole (PPy) and various percentages of poly(ethylene glycol) (PEG) on a TiAlZr electrode, in order to provide antibacterial features. ATR-FTIR analysis highlighted the formation of the PPy–PEG composite film by incorporating PEG into the polymer structure, with more intense PEG infrared absorption peaks when the PEG percentage was raised [59].

ATR-FTIR was also employed to characterize PLGA–PPy[Dex]–Ti samples, developed by Sirivisoot et al., indicating that the OH band, relevant to the intermediate PLGA–NHS, disappeared on PLGA–PPy[Dex]–Ti, thus confirming the reaction of PPyNH_2_ and PLGA–NHS to form the amide (–CO–NH–) bonds in the PLGA–PPy copolymer [38].

Alginate/chitosan coatings electrodeposited on titanium by Wang et al. were also analyzed by ATR-FTIR, proving that alginate and chitosan could be successfully deposited on titanium substrates and form a layer-by-layer structure [39].

The polydopamine coating achieved by GhavamiNejad on the surface of a Ti alloy was confirmed by performing a comparison of ATR-FTIR spectra of the pure Ti alloy and the same coated with polydopamine. The spectrum of pure Ti alloy showed few bands, while the polydopamine coated one showed several new absorbance signals, ascribable to the electrosynthesized polymer [41].

The electrochemically assembled polypyrrole/titania nanotube arrays (PPy/TNTA) coatings on titanium, carried out by Simi et al., were also characterized by ATR-FTIR. The presence of a broad band in the range of 450–800 cm^−1^, observed in TNTA, could be due to the formation of Ti–O–Ti and Ti–O bonds of titanium dioxide. Moreover, the typical polypyrrole absorption peaks in the ATR-FTIR spectrum also confirmed the presence of PPy on TNTA achieved through electrodeposition [43].

Concluding, high interest was recently devoted to ATR-FTIR imaging, in order to obtain spatially-resolved chemical images from different depths within a sample, especially for the analysis of polymer laminates. In the specific case of electrochemistry, an interesting future perspective of ATR can be envisaged in the coupling of in situ ATR with electrochemical systems, in order to supply molecular-level structural information about the electrochemical interfaces [60].

## 3. Surface Topography/Morphology

### 3.1. Atomic Force Microscopy

Despite its short history, atomic force microscopy (AFM) has become one of the essential tools for surface imaging at nanoscale, in particular for biomaterials research. The easy sample preparation for AFM, the opportunity to obtain three-dimensional maps, rather than two-dimensional images, together with the less expensive and time-consuming analysis, allow AFM to be preferred to other imaging techniques, such as SEM or TEM. For example, AFM can provide a direct view of high-resolution surface features also for non-conducting samples, whereas in SEM, a conductive coating must cover insulating materials, and often this process may hide some surface details or alter the sample surface features. Furthermore, AFM analysis does not require vacuum and samples can be imaged at room temperature, in air or liquid phase. In the biomaterials field, these peculiarities make AFM suitable for the characterization of implant surfaces in their native environment and in real time [31,33,34,55,61,62,63]. Furthermore, AFM analysis can be carried out in three modes, i.e., contact mode, non-contact mode, and tapping mode. Different works have documented the importance of this technique in the orthopedic field [64], and in particular for the analysis of titanium surfaces [65]. Albrektsson et al. pioneered the idea of a role of AFM studies for surface implant topography on biological response and bone formation [66].

As far as the electrodeposited coatings on titanium are concerned, different papers reported AFM as an important tool for coating characterization. Several works are related to the electrochemical growth of hydroxyapatite, or hydroxyapatite-like coatings, on titanium and report an accurate AFM imaging [67,68,69]. More specifically, referring to polymeric films electrodeposited on titanium, Manara and coworkers carried out an electrochemically-assisted deposition on titanium of a biomimetic coating, based on self-assembled collagen fibrils and carbonate hydroxyapatite nanocrystals. From AFM surface analyses, an average roughness of about 50 nm height was measured [70].

Pirvu et al. focused their work on the electrochemical deposition of polypyrrole/poly(styrene sulphonate) composite coatings on a Ti6Al7Nb alloy. Different concentrations of the anionic surfactant NaPSS (poli(sodium-4stryrensulfonate)) were used for polymerization. AFM images of the PPy/NaPSS surface appeared to be composed of grains of bigger size, depending on NaPSS concentration, demonstrating that the surfactant concentration plays an important role in the electropolymerization process. Moreover, the surfactant increased the roughness values of the composite with respect to the bare PPy film [71].

Popescu et al. electrosynthesized polypyrrole–polyethylene glycol (PPy–PEG) films on a titanium electrode and contact mode AFM was performed in order to investigate the surface topography of the modified substrate. Small grains of about 0.75 μm and 0.5 μm were observed on PPy and PPy–PEG coatings, respectively. The presence of PEG in the polymerization solution led to a reduction of dimensions of PPy cauliflower-like grains. Moreover, the roughness also decreased with the addition of PEG [72]. Similar conclusions were reported by Ungureanu et al., who performed an AFM analysis of PPy–PEG composite films. Indeed, a granular structure for PPy film without PEG was observed, while grain sizes decreased with the addition of PEG, resulting in a denser composite layer; furthermore, the roughness decreased with the increase in PEG content [59].

**Figure 3:**
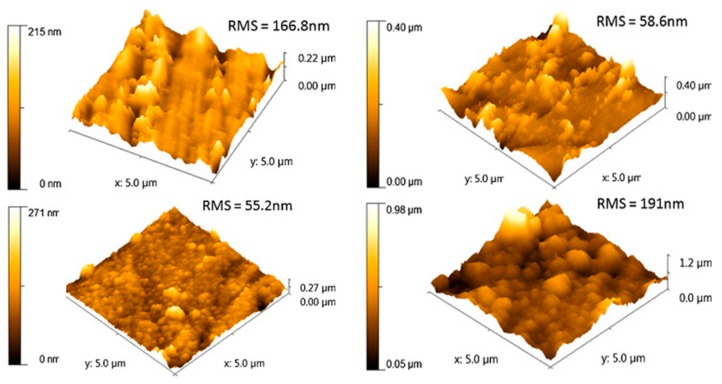
3D atomic force microscopy (AFM) images for Ti/24PDA and Ti/72PDA. Below, Ti/24PDA–PPy and Ti/72PDA–PPy. (Reprinted with permissions from [73], Elsevier 2014 copyright n. 4710121133177).

Another paper of Popescu’s group reported the poly(dopamine) (PDA)-assisted deposition of PPy on Ti substrates. The preparation procedure contemplated firstly the immersion of titanium sheets in a dopamine solution for different times. Then, the PPy films were electrochemically deposited on Ti or on the poly(dopamine)-coated Ti surfaces. AFM analysis confirmed the chemical deposition of PDA, after 24 h of titanium immersion in dopamine solution. When the PPy film was electrodeposited over the PDA coating, the surface became more uniform and the roughness decreased. The PPy was mainly deposited in the spaces between PDA aggregates, forming a compact and uniform PDA–PPy coating. Extending the immersion times in dopamine solution, coverage of titanium surface by PDA was almost complete. AFM measurements demonstrated that the successive electrochemical deposition of polypyrrole on this surface led to the formation of PDA–PPy films with larger grains of PPy, resulting in a final increase of the coating roughness with respect to the PDA–PPy films obtained with shorter immersion times [73] (Figure 3).

According to the aforementioned results, Mindroiu et al. successfully synthesized polypyrrole (PPy) films on a Ti6Al7Nb alloy by potentiostatic polymerization, in the presence of three different surfactants. The structure of the PPy film showed by AFM analysis revealed the presence of well-defined grains, with an approximate diameter of 1 μm. When the surfactants were present, the PPy-based coatings became more compact, since the grain size decreased. Moreover, the obtained Ra values indicated that, adding surfactants, the roughness of the composite films decreased in comparison with that of the bare PPy film. On the other hand, PLGA–PPy[Dex]–Ti samples developed by Sirivisoot and coworkers were analyzed by AFM in non-contact mode, highlighting that cell adhesion was improved by surface roughness. Indeed, PLGA–PPy[Dex]–Ti exhibited the highest Rrms (i.e., (Root Mean Square roughness) among all the samples [74].

Ordikhani and coworkers electrodeposited chitosan–vancomycin composite coatings on titanium implants by a cathodic EDP process. AFM analysis of these coatings revealed that the roughness of the chitosan surface was increased by the embedded vancomycin molecules [75].

The same authors developed electrodeposited graphene oxide/chitosan films with long-term drug-eluting capacity. The characterization of chemically exfoliated graphene oxide nanosheets was also performed by AFM imaging and height profiling, underlying a uniform thickness of around 0.9 nm [76].

In the abovementioned paper of GhavamiNejad et al., the AFM characterization of polydopamine, achieved by dip coating or electropolymerization, was also reported, showing differences in the surface topography of Ti substrates after the two coating procedures. The AFM 3D images clearly evidenced that the polydopamine formed via electropolymerization led to a more homogeneous deposited coating, as compared to that obtained through dip coating [41].

Recently, Bonifacio et al. [30] carried out gallium-modified chitosan/poly(acrylic acid) bilayer coatings on titanium, and they employed AFM to compare the morphology of the poly(acrylic acid) monolayer and the Ga-containing chitosan/poly(acrylic acid) bilayer. AFM images showed that, while the monolayer maintained the surface roughness typical of bare titanium, the gallium-containing bilayer resulted in well-oriented bundle formation and increased roughness.

As highlighted by the above-reported examples, AFM is a fundamental analytical technique for electro-assisted polymers, since its 3D nature allows the calculation of changes in roughness and surface area variations due to differences in deposition parameters.

The last frontier of AFM applied in general to biomaterials and, in particular, to polymer films could be the high-speed AFM, a fascinating technique that allows a spatiotemporally resolved visualization of biomolecules in dynamic action. However, that much of the future of AFM could be related to its coupling with other characterization techniques (hyphenated techniques such as AFM/FTIR, AFM/optical microscopy).

### 3.2. Scanning Electron Microscopy with Energy-Dispersive X-ray Analysis

Scanning electron microscopy (SEM) is an imaging technique based on the signals of backscattered and secondary electrons derived from surface scanning with a finely-focused electron beam. The emitted electrons generate a grayscale image of the sample at very high magnifications. Therefore, SEM is commonly used to study biomaterials’ surface morphology and their cellular response [77]. In particular, SEM is an excellent method to determine the biocompatibility of orthopedic and orthodontic biomaterials, due to the opportunity to observe the surface roughness, as well as the homogeneity of surface coatings. This technique also has the potential to display the interface between a biomaterial and the surrounding biological matter. Sample preparation for SEM analysis involves fixation (for proteins, cells, or tissues), drying, attachment to a metallic stub, and then metallization by sputtering with gold, platinum, or a gold/palladium alloy. It is worth noting that these sample preparation processes might alter the surface morphology of polymeric coatings, particularly those surfaces that may undergo changes in a hydrated environment. Indeed, conventional SEM requires a vacuum. However, SEM can be also carried out in environmental conditions, i.e., environmental scanning electron microscopy (ESEM), a very useful technique in biomaterials science, which provides the opportunity to observe wet or non-conductive samples. The minimal sample preparation represents the main advantages of ESEM. Indeed, the dehydration and metallization steps can be avoided in ESEM mode, allowing the morphology of cells to be assessed at high-resolution in a state closer to their natural morphology in vitro. Furthermore, hydrogels, which are highly hydrophilic crosslinked polymeric networks, could be observed in their wet state by ESEM. In addition, drug delivery systems such as micelles, nanofibers, and liposomes can be easily analyzed by ESEM [78,79,80].

Besides the secondary electrons emitted due to primary electron beam surface irradiation, element-specific X-rays are generated. The combination of SEM with energy-dispersive X-ray (EDX) analysis provides elemental analysis on areas as small as nanometers in diameter.

Most of the aforementioned works reported SEM analysis, together with the other characterizations, in order to gain insights into the morphology of titanium polymeric coatings obtained by electrochemical routes [34,51,53,54,55,56,57,58,61,62,63,81].

For example, Erakovic et al. reported the SEM–EDX characterization of lignin, hydroxyapatite, and the HA/Lig coatings deposited on titanium. In particular, the homogenous surface, without fractures, of the composite coating indicated that lignin strengthened the bonding between HA particles and the substrate surface [32]. In another work, the same authors reported an SEM investigation of silver-loaded hydroxyapatite/lignin coatings electrodeposited on titanium. SEM micrographs of Ag/HA/Lig revealed the surface homogeneity of the coating before immersion in SBF. Conversely, after immersion in SBF, the presence of a crystalline surface was observed [37].

Moreover, the antibacterial polypyrrole and polyethylene glycol coating on a TiAlZr developed by Ungureanu et al. was additionally studied by SEM in their topographical features. In agreement with the abovementioned AFM conclusions, SEM demonstrated that the polymeric coating without PEG presented a uniform distribution of both nanometric and micrometric grains, with a porous structure of cauliflower morphology. On the other hand, the PEG introduction during polymerization, up to 2%, led to a reduction in grain size and a higher covering degree, while maintaining the cauliflower morphology of the coating. For the film obtained in the presence of 4% PEG, larger grain sizes were evident, even if the film remained compact [59].

In order to describe different alginate and chitosan layers deposited on a titanium substrate, Wang et al. supplied the SEM fracture surface of the layer-by-layer coatings, which allowed them to estimate the thickness of alginate and chitosan coatings and to speculate on the reason for the higher chitosan layer thickness, as compared to the alginate one [39].

Similarly, Popescu and coworkers analyzed their PDA–PPy coatings on titanium by SEM, observing differences in surface features after the deposition of PDA for 24 or 72 h (coded as 24PDA and 72PDA). After the deposition of 24PDA or 72PDA on titanium, the electrodeposition of PPy was also studied in terms of surface morphology. While on the Ti/24PDA surface the PPy film grew uniformly, on the Ti/72PDA surface the PPy film polymerized, forming bigger clusters [72].

The surface morphology of the dip-coated and electropolymerized poly(dopamine) coatings developed by GhavamiNejad et al. was further observed by means of FESEM. The latter differs from conventional SEM in the type of sample irradiation. Indeed, rather than a thermoionic emitter, an FESEM is equipped with a field emitter gun, also known as a cold cathode, which provides a narrow beam, thus enabling the enhancement of the spatial resolution at high and low electron energy. By means of FESEM, GhavamiNejad et al. reported that the dip coating procedure led to agglomerated particles and high surface roughness, with a subsequent uncontrollable self-crosslinking. Furthermore, this inhomogeneity caused the agglomeration of silver nanoparticles after reduction by the catechol groups. In contrast, the electropolymerized coatings exhibited a relatively smoother coating, leading to a uniform distribution of AgNPs [41].

**Figure 4:**
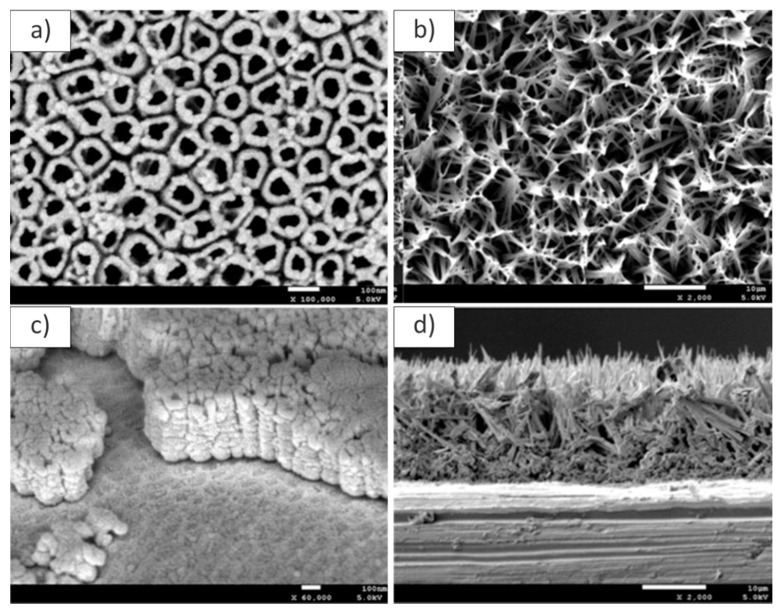
(**a**) FESEM image of TiO_2_ nanotubes and (**b**) GelGOHA composite coating on titanium. (**c**) Cross-section of TiO_2_ nanotubes and (**d**) GelGOHA coating on titanium. (Reprinted with permission from [40], Elsevier 2015 copyright n. 4710121336953).

Yan et al. developed a titanium coating made of graphene oxide cross-linked gelatin fillers (GelGO), dispersed in hydroxyapatite (HA) on TiO_2_ nanotube arrays (TNs). The authors analyzed the prepared coating by SEM–EDX, observing that TNs grew highly ordered and in a vertical direction from the Ti substrate (Figure 4). Moreover, HA-coated substrates exhibited the typical needle-like crystals, whereas the GelGOHA coatings presented nest-like pores, more favorable for cell adhesion and mass transport. In addition, the EDX of the GelGOHA coating revealed a ratio of Ca/P lower than the ratio typical of human native apatite [40].

Finally, Simi et al. also employed SEM analyses to characterize electrochemically assembled PPy/TNTA coatings on Ti. In this case, as in the aforementioned, titania nanotubes were highly ordered and aligned vertically on the titanium surface. In the presence of PPy, the nanotube diameter decreased, while wall thickness increased. Moreover, PPy initially filled the intertubular spaces between the nanotubes, then covered the nanotubes’ surface. The cross-sectional images showed a PPy deposition throughout the length of the nanotubes [43].

Even if, concerning thin films, SEM and AFM might produce a similar representation of the sample surface—for SEM, a large area observation (up to several millimeters) of the variations in surface morphology can be acquired in a single scan, whereas a 100 µm × 100 µm area is typically the largest area sampled by AFM.

Moreover, the conventional SEM–EDX coupling, using X-ray and backscattered electrons, simultaneously supplying elemental analysis and morphological mapping, represents the main advantage of this technique nowadays, also for electro-assisted polymer coatings analysis. However, different from XPS analysis, no information about the speciation or oxidation state of each element could be supplied by EDX. On the other hand, EDX supplies quantitative information, while XPS and TOF-SIMS are both semi-quantitative techniques.

## 4. Surface Phenomena

### 4.1. Ellipsometry

Ellipsometry is a sensitive optical technique that measures changes in the state of light polarization upon specular reflection from a planar interface. This technique represents not only a simple tool for the metrology of film thickness, but it allows also to obtain information about the organic interfacial structures [82].

Ellipsometry has many applications in different fields, such as semiconductor physics, microelectronics, and life sciences. In the biomaterials field, ellipsometry is commonly used to characterize the thickness of polymeric or inorganic layers on the substrates, ranging from a few Angstroms to several micrometers, for layers which are optically homogeneous and isotropic and when a significant refractive index discontinuity exists at the interface. Thanks to its non-destructive and contactless features, this technique appears particularly desirable in the evaluation of soft layers. Indeed, different reviews have reported the usefulness of ellipsometry for studies of specific processes, such as swelling of thin polymer films [83], and protein absorption on metal oxide coatings for biomedical implants [84].

Concerning thin polymer coatings electrodeposited on titanium substrates, a few but significant works reported ellipsometric results [85]. In particular, Tanaka and coworkers carried out the electrodeposition of poly(ethylene glycol) on a titanium surface, terminated at one or both ends with amine bases, to prevent the adsorption of proteins. The thickness of the deposited PEG layers, obtained by immersing titanium in a PEG solution or electrodepositing PEG on Ti, was evaluated using ellipsometry. The obtained results indicated that electrodeposition was more effective than immersion to coat a titanium surface with PEG [86,87]. Similarly, Oya and Tanaka et al. investigated the effect of RGD peptide, immobilized through the electrodeposition of PEG or glycine (Gly) on titanium, on the calcification led by MC3T3-E1 cells. The thickness of all the immobilized layers on Ti was determined by ellipsometry. Before RGD immobilization, no differences in the thicknesses of PEG/Ti and Gly/Ti layers were observed. Therefore, the immobilized number of molecules is larger in Gly/Ti than in PEG/Ti, likely because Gly molecules are much smaller, and more easily electrodeposited on Ti than PEG. After RGD immobilization, the thickness of all specimens increased, as expected. Since the thicknesses of RGD/PEG/Ti and RGD/Gly/Ti specimens were similar, Oya et al. concluded that more RGD molecules were immobilized on PEG/Ti than on Gly/Ti [88].

Following previous studies, this research group proposed RGD immobilization through electrodeposited PEG on titanium dental implants. The aim of the work was in vivo bone healing enhancement, achieved through early osteoblastic cell attachment and subsequent differentiation, promoted by integrin binding. The thicknesses of PEG and RGD/PEG layers immobilized on Ti samples were determined by ellipsometry [27]. Finally, they evaluated the biofilm formation, in the presence of saliva, on PEG-deposited titanium. The thickness of the immobilized PEG layer, measured by ellipsometry [89], was equal to 2.49 ± 0.39 nm.

### 4.2. Contact Angle

The initial contact and interaction of implants with surrounding living tissues starts with protein adsorption on the implant surface. In this process, surface properties, like wettability and surface tension, are critical to the biocompatibility of the implanted materials and can be crucial criteria for biomaterial selection, in particular for orthopedic and orthodontic implants. For titanium and titanium alloys, a deep influence of surface roughness on surface wettability and, in turn, on the biological response at the implant/bone or implant/soft tissue interfaces, was ascertained [90,91]. Wettability depends on two main surface characteristics: surface chemistry and surface topography. Several common approaches for the analysis of surface wettability have been adapted to study titanium implant surfaces.

The hydrophilicity or hydrophobicity nature of a surface is a relatively important property in the characterization of the surface energy of biomaterials, as these properties can affect the amounts and types of bound proteins, as well as cellular responses and subsequent tissue reactions. Different works evidenced that hydrophilic surfaces improved the early stages of cell adhesion, proliferation, differentiation, and bone mineralization compared to hydrophobic surfaces [92,93]. However, opposite results have been found in other studies [94,95]. Moreover, hydrophilicity/hydrophobicity also influences bacterial colonization, a serious issue of concern in dentistry. In principle, hydrophobic and hydrophilic bacterial strains will preferentially adhere to surfaces with similar affinities to water [96].

To gain insights into the wetting behaviour of a given biomaterial, the sessile drop, or contact angle (CA) technique, is commonly used. In this analysis, a drop of the desired wetting liquid (e.g., water) is placed on the biomaterial surface, and the angle between the solid surface baseline and the tangent of the drop at the solid/liquid/gas boundary is measured.

**Figure 5:**
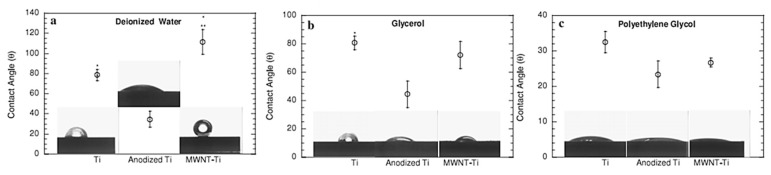
Contact angle measurements with three liquids using an EasyDrop model system (Kr¨uss): (**a**) double-deionized water, (**b**) glycerol, and (**c**) polyethylene glycol (PEG), all on conventional pure Ti, anodized Ti, and MWNT–Ti. Data = mean ± SEM; *n* = 3; * *p* < 0.01 compared to anodized Ti and ** *p* < 0.01 compared to Ti. (Reprinted with permissions from [38], IOP Publishing Group 2011 copyright n. 1003986).

Since titanium or titanium alloys’ surface wettability is important, it is intuitive that this property represents a crucial parameter in the choice of the most suitable polymer coating, which covers the metal surface, therefore changing its hydrophilic or hydrophobic nature. As far as the electrodeposited polymer films on titanium are concerned, CA is often employed in combination with most of the abovementioned surface analysis techniques [31,38,53,57,63,81,97] (Figure 5). For example, in the case of polypyrrole/poly(styrene sulphonate) composite coatings electrodeposited on Ti6Al7Nb alloy, Pirvu et al. demonstrated that wettability can be controlled by changing the pyrrole:poly(styrene sulphonate) polymerization ratio. Moreover, the surfactant concentration showed a great influence on surface wettability [65].

In the case of coatings, electrodeposited on a Ti alloy, based on PPy and various percentages of PEG, it was demonstrated by CA measurements that wettability is altered by different PEG concentrations [59]. Moreover, Cometa et al. carried out the electrosynthesis of novel antimicrobial coatings on metal devices, based on poly(ethylene glycol diacrylate) (PEGDA) hydrogel thin films embedding copper-based nanoparticles. Coated surfaces were very hydrophilic and the addition of copper nanoparticles did not significantly alter the hydrophilic nature of PEGDA coating [98].

PPy films electrosynthesized on Ti6Al7Nb alloy by Mindroiu et al. in the presence of three different surfactants were also evaluated by contact angle measurements. The surface wettability was greatly influenced by the type of surfactant added. Indeed, the highest hydrophilic character was observed for PPy/PSS films, with respect to PPy/Triton X-100 and PPy/DM ones [74].

PDA-assisted deposition of PPy on Ti substrates, carried out by Popescu and coworkers, was also analyzed by CA analysis. After PDA functionalization, dramatic changes in contact angle values were detected, indicating that the contact angle values of the composite films were intermediate between pure PDA and PPy films [73].

Fukuhara and coworkers carried out an interesting work in which the usefulness of the CA measurements was noteworthy. These authors electrodeposited a polymer composed of a poly(2-(methacryloyloxy)ethyl phosphorylcholine (MPC)) segment and a poly(2-aminoethylmethacrylate) segment on titanium, in order to inhibit platelet adhesion. Thanks to this surface modification, the water contact angle decreased and, as a consequence, the amount of adsorbed protein on the modified surface decreased, reducing clotting [99].

Interestingly, Buxadera-Palomero et al. compared three methods for the synthesis of polyethylene glycol (PEG) coatings on titanium, i.e., plasma polymerization, electrodeposition, and silanization. Thanks to CA analysis, the authors concluded that plasma coating led to significantly more hydrophilic samples, with the highest wettability. On the other hand, silanized and electrodeposited PEG presented approximately the same hydrophilicity. The authors explained these lower contact angle values for the plasma-polymerized sample with the formation of oxygen functionalities in the polymer, thanks to the plasma process [100].

Finally, Simi and coworkers characterized their electrochemically assembled polypyrrole/titania nanotube arrays (PPy/TNTA) on titanium sheets by CA measurements. The water contact angle of the TNTA specimen was lower than that of the titanium substrate, demonstrating the hydrophilic nature of the nanotubes. The water contact angle obtained for the PPy/TNTA specimen was intermediate between that of TNTA and that of pure Ti. The authors ascribed this slight hydrophobicity increase of TNTA to the partial filling of PPy into and in between the nanotube arrays, as revealed by SEM. However, the PPy/TNTA specimen still exhibited a hydrophilic character, due to the presence of PPy amine groups [43].

In conclusion, among the more modern surface analytical tools for wettability estimation, CA remains the standard and easiest method to measure hydrophilicity/hydrophobicity of thin polymer films, even if samples with high surface roughness, irregular surfaces, or highly water-absorbing polymers could not be analyzed with this technique.

### 4.3. Quartz Crystal Microbalance Dissipation Monitoring

The quartz crystal microbalance (QCM) is a high-resolution mass sensing technique for analytical chemistry and electrochemistry applications, based upon the piezoelectric effect. It is able to detect mass variations due to monolayer surface coverage, starting from small molecules to polymer films, complex arrays of biopolymers and biomacromolecules, up to whole cells. In addition, the QCM, in the dissipation monitoring configuration (QCM-D), can provide information about the energy-dissipating properties of the bound surface mass. The QCM-D detectable systems are micelles, self-assembling monolayers, molecularly imprinted polymers, chemical sensors, layer-by-layer assembled films, and electroactive polymer films. This technique can also describe the attachment strategies of polymer brushes on metal substrates or grafting reactions of molecules on polymer pendant groups. Moreover, QCM-D allows the study of complex biochemical and biomimetic systems, designing protein, antibodies, nucleic acids, or whole cells biosensors [101].

Another important and unique feature of this technique is the ability to measure the in situ mass and energy dissipation properties of films, while simultaneously carrying out electrochemical processes, such as, for example, the electropolymerization of a film on an oscillating quartz crystal electrode (E-QCM) [102].

Kasemo and co-workers pioneered the use of QCM-D to study biological surfaces [103]. In the orthopedic sector, they studied the adsorption kinetics of three model proteins (i.e., human serum albumin, fibrinogen, and hemoglobin) on titanium dioxide surfaces, using this technique combined with ellipsometry and optical waveguide light mode spectroscopy [104]. Moreover, the same group described a method to coat a gold quartz crystal microbalance with dissipation sensor with an ultra-thin layer of hydroxyapatite. The authors studied the in situ adsorption mechanism and conformational change of fibrinogen on gold, titanium, and hydroxyapatite surfaces by QCM-D [105].

Regarding the electrodeposition of polymers on titanium substrates, Lassalle and coworkers described in 2001 the synthesis of a new pyrrole–oligonucleotide building block, electropolymerizable on crystal microbalance electrodes to obtain biosensors for DNA hybridization detection in real time, through micro-gravimetric transduction. The electrodes were coated on both sides of the quartz by successive sputtering of titanium and platinum. This work demonstrated the ability of this technique to reach a direct and quantitative detection of DNA, even if an enhancement in the sensitivity and accuracy of the QCM response became necessary [106].

**Figure 6:**
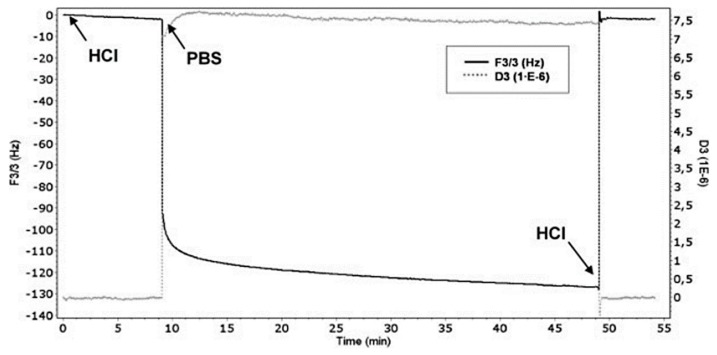
QCM-D study of the swelling–deswelling of PEGDA-co-AA hydrogel coating at different pH solutions. Arrows and labels indicate injections of HCl (pH 2.2) solution, PBS (pH 7.4), and the final HCl (pH 2.2) solution, respectively. (Reprinted with permission from [25], Whiley 2009 copyright n. 4710141377265).

De Giglio et al. proposed a new electrochemical polymerization method to develop polyacrylates-based hydrogel thin films onto metal substrates, with interesting potential in the orthopedic field for in situ drug delivery applications. In particular, QCM-D was used to evaluate the pH-dependency of the swelling process of these hydrogels [25] (Figure 6). This research group also developed the electrosynthesis of PAA films to protect metal orthopedic implants against corrosion. The swelling of PAA seemed to have a crucial role in the barrier performances of these films and this process, in turn, exhibited dramatic changes depending on pH modifications. In this respect, the authors employed QCM-D to monitor the swelling behaviour of the PAA coatings in different pH solutions. The QCM-D experiments confirmed that the swelling phenomenon hugely influenced the barrier behaviour of PAA coatings, as well as its electrostatic interaction with ions [23].

Moreover, De Giglio et al. addressed a paper on the study of different solid–liquid interfacial processes related to poly(2-hydroxyethyl methacrylate) (PHEMA)-based thin coatings electrosynthesized on quartz crystal electrodes. In the work, they proposed these hydrogels as bioactive titanium implant coatings or controlled release devices (e.g., stents or microchips). QCM-D was employed to investigate the correlation between the swelling behavior and the thickness of the electrosynthesized films [107].

QCM-D analysis is often carried out together with ellipsometry. Indeed, both techniques provide information about adsorption events on surfaces, quantifying adsorbed masses in real-time. The use of both techniques makes it possible to distinguish features such as film swelling or collapse from adsorption/desorption events or other structural or morphological changes of adsorbed films. On the other hand, the peculiar information provided by QCM-D and ellipsometry consist of the viscoelasticity properties of thin films and optical film features, respectively.

## 5. Mechanical Characterization

### 5.1. Nano- and Micro-Indentation

Titanium has been largely used as a material in biomedical technology and implant applications for its appropriate mechanical properties; however, a substantial difference between the elastic modulus (E) of the human bones and the titanium can cause a possible early failure of the implant [61]. This implies an additional phenomenon, called stress shielding [108]. Indeed, if the loads on the implant, which come from patients’ weight and their activities, are not homogeneously distributed to the bone, the implant may present different zones with localized higher stresses. Conversely, under stress, the bone density may reduce for adapting to the new under-stress situation (osteopenia) [109]. In order to avoid the mismatch between native bone and implant, recently, several studies have focused on a combination of the metallic implants with polymeric coatings, which shows important enhancements to reduce the stress shielding effect [110,111,112]. Therefore, understanding the resultant mechanical properties of the polymeric-coated implants may allow the prediction of the implant’s in vivo response.

**Figure 7:**
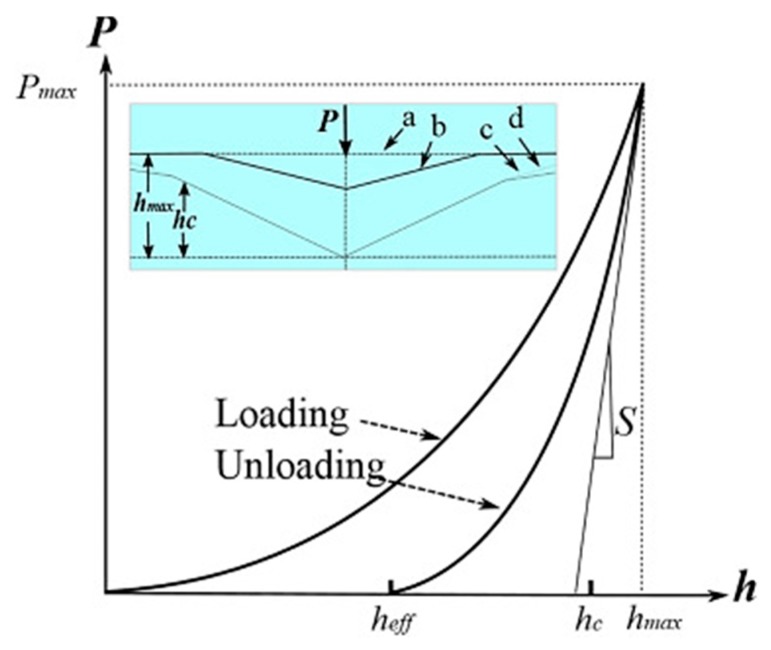
A Schematic representation of a load–displacement curve in indentation testing. (**a**) Initial surface; (**b**) surface after load removal; (**c**) indenter; (**d**) surface profile under load. *P* is the peak indentation load; *h_max_* is the indenter displacement at peak load; *h_eff_* is the final depth of contact impression after unloading; and *S* is the initial unloading stiffness. (Reprinted with permission from [113], Materials Research Society 1992 copyright n. 4710150228702).

In this respect, nano- and micro- indentation methodology has been largely employed to obtain a microscopic evaluation of the mechanical properties of polymeric coatings, including elastic modulus and hardness, with high precision and accuracy [113]. In particular, during the complete cycle of loading and unloading of the cantilever (as shown in Figure 7), a force–displacement curve is plotted by measuring the deflection of the cantilever towards and away from the specimen. The elastic modulus and the hardness can be measured from the force–displacement curve, considering the specific relationships between the applied force and the resultant depth of the indentation (displacement). According to the Oliver–Pharr model [114], the hardness (H) can be determined as the indentation load, divided by the resultant contact area of the indentation (A_p_), using the following equation:

H = P_max_/A_p_, (1)

where P_max_ is the maximum applied load, measured at the maximum depth of indentation (h_max_).

On the other hand, the effective elastic modulus E_eff_ can be calculated by:

(2) $$E_{eff}=\frac{1}{\beta }\;\frac{\surd \pi }{2}\;\frac{s}{\sqrt{A_{p}}},$$

in which *β* is a constant depending on the indenter geometry (e.g., for a Berkovich indenter *β* is equivalent to 1.034) and *s* the slope of the curve upon unloading, which is indicative of the stiffness of the contact. This value generally includes a contribution from both the materials being tested and the response of the test device itself. Furthermore, the effective elastic modulus is correlated with the elastic modulus of the sample by:

(3) $$\frac{1}{E_{eff}}=\frac{1-v^{2}}{E}+\;\frac{1-v_{i}^{2}}{E_{i}},$$

where *E* and *v* are the elastic modulus and the Poisson’s ratio of the sample, respectively. *E_i_* and *v*_i_ are the same parameters for the indenter. As an example, for a diamond indenter *E_i_* is 1140 GPa and *v*_i_ is 0.07.

A recent application of indentation measurement was proposed by Bosh et al., who investigated the mechanical properties at the nanoscale of a polymeric coating on a titanium implant, consisting of a semi-crystalline ethylene chlorotrifluoroethylene (ECTFE) fluoropolymer, called Halar^®^. This material is currently considered one of the most chemical-resistant polymers available on the market [60], and it can represent a suitable biomaterial for coating applications, due to its elastic modulus close to the native bone (ranging from a few to 30 GPa) [115]. As mentioned above, this range of the E value can potentially avoid the stress shielding effect. Furthermore, Bosh employed the EDP for the manufacturing of the Halar^®^ coating, providing a hard and tough layer to the implant, as well as outstanding additional properties for a bio-coating (i.e., abrasion resistance, biological stability, low surface energy, and coefficient of friction) [116]. Nanoindentation outcomes revealed values of hardness and elastic modulus (by taking the average of five data points) of 46.8 ± 0.4 MPa and 1.2 ± 0.7 GPa, respectively, slightly lower than the one reported for native bone (5–20 GPa). Furthermore, other synthetic polymers, such as Polyetheretherketone (PEEK) can be easily electrophoretically deposited on a Ti alloy for improving bio-tribological application (e.g., wear resistance, corrosion resistance in Ringer’s solution) as well as the mechanical properties, as reported by Sak et al. [117].

Although the use of synthetic polymers can allow the creation of a stable and hard coating, the use of “soft” natural-based polymers to modify the surface of “hard” metallic implants gained a lot of attention for their excellent in vivo responses, such as mineral phase deposition, osteoconductivity, and osteoinductivity [118]. As natural polymers, chitosan (CH), silk fibroin (SF), and gelatin (GL) have become well-known biopolymers, due to the fact that they are low-cost and abundant in nature, strongly biocompatible with low (or completely absent) immunogenicity, and with tunable physical and mechanical properties [119,120,121]. Guo et al. reported an elastic modulus of an SF-coated Ti-based implant of 4.5–7.0 GPa, compared with 110 GPa of the bare Ti sample [120]. Although there are significant differences between the metallic implants, the measured E value for this ~15 µm thick SF coating was close to the human bone. Furthermore, the presence of SF improved the viability and proliferation of MG63 osteoblast-like cells.

An alternative approach to improve the in vivo response concerns the electrochemically-assisted deposition of biomimetic composite coatings on titanium plates, in order to further improve the implant osteointegration [34]. The electrolytic processes can be easily carried out using a type I collagen suspension in diluted solutions, which contain the precursors for the nucleation of hydroxyapatite (HA) crystals (such as Ca(NO_3_)_2_ and NH_4_H_2_PO_4_). The process can be performed in mild conditions (room temperature and neutral pH), applying a constant current for different periods of time [69]. A similar procedure was proposed by Murugan et al., who electrodeposited an innovative, multi-ion (Zn^2+^ and Mg^2+^)-loaded HA coating, topped with a Poly(ε-caprolactone)/graphene oxide (PCL/GO) layer on a Ti alloy. This hybrid material was intended to provide higher biocompatibility, mechanical, and antibacterial properties. Furthermore, the porous-like structure of the composite coating could contribute to the growth and integration of bone. The indentation tests revealed that the combination of PCL, HA, and GO dramatically improved the mechanical properties, in terms of hardness, that can be due to the presence of GO, which contains epoxide and hydroxyl groups on the basal plane, as well as carboxyl and carbonyl moieties on its edges [122].

Finally, the indentation-based technique is suitable for several standard materials used in engineering and some hard biomaterials, such as bone; however, for the materials characterized by time-independent mechanical responses (e.g., soft coated biomaterials), the Oliver–Pharr method is not valid, because a negative or vertical slope can occur in the initial unloading area [123]. Another drawback of the Oliver–Pharr method is related to the contact area, which can lead to substantial errors if the “sink-in” or “tip radius” effects are present [124]. Therefore, correction factors are required to evaluate the actual properties of the material, e.g., high unloading rates or long hold periods.

### 5.2. Peel (or Adhesion) Tests

**Figure 8:**
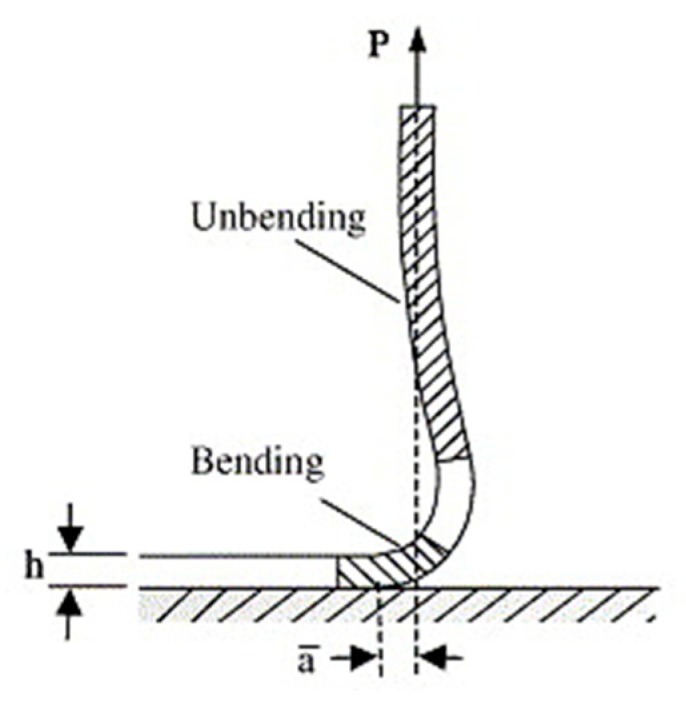
Peel test mechanism. (Adapted and reprinted with permission from [125], Elsevier 2005 copyright n. 4710150365138).

In order to measure the adhesion strength of electro-assisted polymeric coatings on Ti implants, the peel (or adhesion) tests are largely reported in literature. This mechanical characterization method is performed to evaluate the resistance between the coating and the substrate to highly localized stresses [125,126], where peel forces are applied to linear fronts (Figure 8. When the adhesive is characterized by a higher modulus and the adherend is more flexible, the stressed area is reduced to linearity and, then, the stress approaches infinity. Moreover, the area over the stress applied is dependent on the thickness and modulus of adhesive, and it becomes very difficult to measure precisely. Therefore, the applied and failing stress can be described also as linear values, i.e., force per linear cm, in addition to force per area.

Murugan et al. performed the adhesion strength tests according to ASTM F 1044-05, recommended for ceramic or composite coatings on metallic implants. In particular, all the coated samples were kept in an oven at 100 °C for 1 h before the tests and the fixtures were subjected to the pull-out test at a crosshead speed of 1 mm/min. In this work, the authors demonstrated a high adhesion strength of the coated PCL/GO/HA composite (29.6 ± 0.4 MPa), suitable for high load bearing applications. Interestingly, they reported that low concentrations of GO improved the mechanical properties of HA/PCL-based composite coatings [122].

Depending on the material that constitutes the coating, different ASTM protocols can be employed. Jacques et al. considered ASTM D3359 in order to evaluate the adhesion of an electro-grafted organic layer, based on 4-pyrrolyphenyldiazonium (Py-PD) on NiTi substrates. This test consisted of scratching the modified sample with a metallic comb, with spacing streaks of 1 mm; then, a scotch-tape was stuck on the scratched sample and removed [36]. Conversely, ASTM D4541-02 procedure E was followed by Simi et al. in order to evaluate the adhesion strength of a polypyrrole/Titania nanotube array (PPy/TNTA) hybrid coating on a Ti implant [43]. This standard test method is designed to measure the pull-off strength of coatings by using portable adhesion testers.

Although different ASTM standard procedures are available for the peel tests, considering the diverse nature of the implied components, the reliability of the peel test can be limited by the process parameters. Indeed, in addition to the coating thickness and modulus, the peel rate and the angle of peeling are other factors that can play a crucial role in the evaluation of the mechanical properties. In their work, Bush et al. considered a 90° peel test, and the coating was peeled at a slow uniform speed, in order to determine the adhesion strength of the Halar^®^ polymer coating on the Ti substrate [61]. Furthermore, the peel test is limited to thin flexible adherends and the excessive extension of flexible adherends can lead to failure at the interface between adhesive and adherend.

### 5.3. Fatigue Tests

Finally, for load-bearing applications, the fatigue tests are suitable for evaluating the lifespan of material where the fatigue life is defined as the total number of cycles that a material can be subjected to under a single loading scheme. Moreover, a fatigue test is used for measuring the maximum load that a sample can withstand for a specified number of cycles. Among several common types of fatigue testing, the three-point bending test is used in the ISO standard to determine the mechanical strength of dental polymeric (or composite) dental implants, while the biaxial flexural and four-point bending tests are suggested in the ISO standards for ceramics-based dental implants. The three-point bending tests can be performed under a sinusoidal constant amplitude load, at different frequencies (usually ranging from a few to 10 Hz). In this test, a flat sample is placed on two parallel supporting plates with the coating facing down. The loading force F is applied in the middle of the sample. Stresses in the positive direction represent tensile stresses, leading to material contraction. In particular, for a sample characterized by a rectangular cross section, the maximum stress at a three-point bend can be calculated by:

(4) $$\sigma_{max}=\frac{3FL}{2bh^{2}},$$

where *h* and *b* are the thickness and width of the sample respectively, and *L* is the distance between the parallel plates.

**Figure 9:**
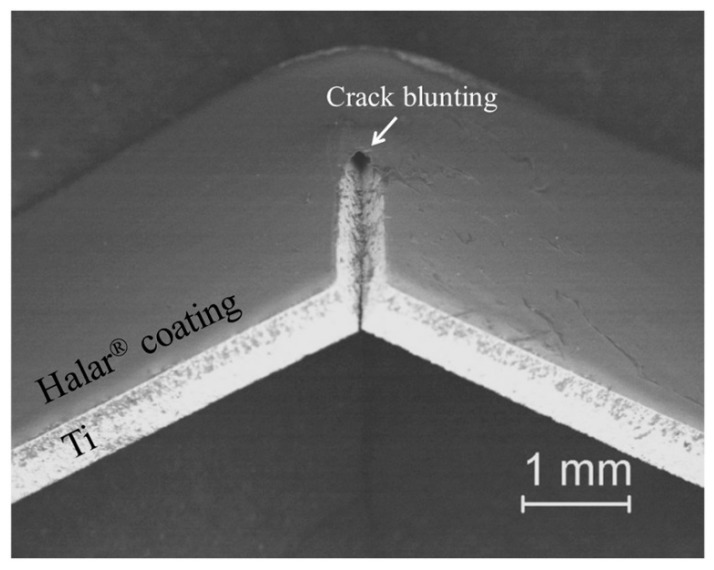
SEM micrograph of the crack growth in a Halar^®^-coated Ti specimen. (Reprinted with permission from [61], Elsevier 2018 copyright n. 4710150493083).

According to ASTM E855, Bush et al. performed fatigue tests in order to evaluate the fatigue strength versus the number of cycles up to failure of uncoated Ti- and Halar^®^-coated implants [61]. Usually 10^7^ load cycles are largely accepted as the fatigue limit to predict the formation of cracks in the coating and the bulk material. In general, the fatigue fracture is the result of a combination of intrinsic (damage) factors ahead of the crack tip and extrinsic (shielding) factors, mainly behind the tip trying to impede it. From this test, an increase in fatigue strength was observed in the coated samples, compared with the uncoated ones. A following inspection of the crack tip in the polymeric coating evidenced the blunting of the crack, which caused a stable deformation around the crack tip, consequently increasing the depth of the plastic area. Despite the high number of the load cycles, when the coating failed, no detachment of the coating from the Ti implant was evident. The concentrated plastic deformation occurring at the crack tip was a regulatory aspect for the growth of the crack and, therefore, highlights the blunting of the crack tip (Figure 9).

One limitation of the cyclic-fatigue tests concerns the use of very expensive testing machines, where usually only one sample can be tested at a time, causing long-term testing and massive financial costs. Therefore, in order to reduce the experiment time, the use of multi-specimen equipment is highly recommended, because it can allow independent testing of many samples simultaneously at different conditions. Moreover, the correlation between the number of cycles and the years of service is still controversial in the literature. As an example, Rosentritt mentioned that the use of 1.2 million cycles to represent five years of service has been suggested [127]. In contrast, 500,000 cycles was credited by Zahran et al. to simulate 10 years [128].

## 6. Discussion and Conclusions

Over the past two centuries, the synthesis of electro-assisted polymeric coatings has provided new opportunities for the development of smart titanium prostheses, with tunable features, for dentistry and orthopedics. Polymeric coatings support the biomaterial’s osseointegration, prevent microbial colonization, and hinder corrosion. Moreover, the presence of a polymer coating is exploited to obtain stimuli-responsive implants, able to deliver bioactive molecules with predictable kinetics. Controlling the physico-chemical, morphological, and mechanical features of the polymeric coatings it is possible to drive their interactions with the host tissues. Therefore, choosing the right combination of chemical composition, roughness, wettability, topography, and stiffness is an effective strategy to obtain multifunctional implants with the expected biocompatibility. Hence, the accurate characterization of physico-chemical, morphological, and mechanical features of polymeric coatings is essential to improve these systems, reaching the optimized balance between all their relevant features. In this work, the role of classical and advanced techniques to characterize polymeric coatings deposited on titanium by electro-assisted routes was presented, with a focus on the basic principles and most frequent applications.

The potential of spectroscopic methods (i.e., XPS, EDX, ATR-FTIR, TOF-SIMS) in accurately determining the surface composition of titanium coatings was herein reported. In this respect, ATR-FTIR still represents a cheap and rapid technique to gain qualitative insights into the chemical composition of a polymeric coating. In addition, if XPS could be easily exploited to calculate the surface-relative elemental abundance and their chemical environment (oxidation state, functional groups, etc.), by contrast, TOF-SIMS provides information about the presence of particular isotopes. Furthermore, the elemental and molecular distribution on a polymeric surface could be investigated in high resolution by TOF-SIMS. Nevertheless, as far as large area images and elemental mapping are concerned, SEM–EDX represents the most common physico-chemical and morphological analysis performed. Further details, especially on nanotopographical and interfacial features, are provided by AFM. The latter could be exploited to address coating roughness with high resolution, in a fast and cost-effective way. In addition, ellipsometry provides useful data on soft coatings’ thickness, as well as cues on polymeric swelling and protein adsorption. In this respect, a real-time kinetic of molecular adsorption could be studied, combining ellipsometry with QCM-D. The swelling behaviour and adsorption potential of polymeric coatings are tightly linked to their wettability, which is routinely determined by CA measurements. Further insights on the coatings’ resistance to mechanical stressors, in terms of adhesion and fatigue strength, could be measured by peel and fatigue tests, respectively. The evaluation of surface hardness and elastic modulus by means of indentation tests provides further details on the mechanical behaviour of the coated implant, with high precision and accuracy. However, the application of the Oliver–Pharr model cannot be applied in the presence of coated materials characterized by independent mechanical responses, and corrections factors should be carefully considered in the presence of the above-mentioned “tip radius” effect. Furthermore, the evaluation of the adhesion strength of the polymeric-based coating on the medical implant is fundamental to design and predict some biological features, e.g., antibacterial properties of the coating, controlled release of biomolecules from the coating, integration, and stability of the coated implant within the surrounding tissues. Different ASTM standards are accessible for the peel tests; however, several process parameters (e.g., coating thickness and modulus, peeling angle, peel rate) should be carefully planned because they can dramatically influence the reliability of the tests. Finally, fatigue tests are appropriate for measuring the lifespan of the medical implants under specific testing conditions. Whereas correlations between cycles and years of service have been attempted, such basic mathematical translation cannot be supported by current evidence, and remains controversial in literature.

The opportunity and challenges to combine physico-chemical, morphological, and mechanical data, presented in this review, are crucial to improve the knowledge of the synthesized coatings, further advancing their development, ultimately leading to optimized implants. Future research dealing with polymeric coatings should focus on the integration of the most advanced analytical and mechanical techniques to go beyond current limitations of electro-assisted synthetic routes, in order to transfer the development of polymeric coatings into large-scale, engineered processes for the biomedical industry.

## Figures and Tables

**Table 1:** The main analytical and mechanical techniques for coating characterization.

Technique	Measured Parameter/s	Sampling Depth/Height	Information Obtainable	Limitations	References
XPS	Binding energy of electrons	5–10 nm	Elemental composition (qualitative and quantitative), chemical bonds, or oxidation states	Extra-dry state, need for ultra-high vacuum, sensitivity to contamination, lack of hydrogen and helium detection	[17,18,19,20,21,22,23,24,25,26,27,28,29,30,31,32,33,34,35,36,37,38,39,40,41,42,43,44]
TOF-SIMS	Mass/charge	1–2 nm	Type of atoms, molecules, and pendant groups on the surface	Dry state, need for vacuum, sensitivity to contamination, difficult quantification	[20,21,46,47,48,49,50]
ATR-FTIR	Transmittance	500 nm–2 µm	Organic (and some inorganic) material identification, both in liquid and solid state	Need for maximal optical contact between the sample and the IRE (flat surfaces)	[31,37,38,39,41,43,51,52,53,54,55,56,57,58,59,60]
AFM	Force between the probe and the sample	Atomic–few µm	Topography, coverage	Artefacts, contamination.	[41,59,67,68,69,70,71,72,73,74,75,76]
SEM–EDX	Interaction of electron beam with atoms	0.2–2 µm	Detailed high-resolution images, with elemental identification and quantitative compositional information of the analyzed spots	Artifacts due to sample preparation, limited to solid and small samples, need for vacuum	[32,34,37,39,40,41,43,53,63,72,81]
Ellipsometry	Change in polarization of incident radiation interacting with sample	300 nm	Coating thickness, absorption kinetics	Highly model-dependent, need of refractive indices of all layers, assumption of homogenous surfaces	[84,85,86,87,88,89]
CA	Angle between liquid and surface of solid sample	<1 nm	Surface free energy, wettability	Contamination	[31,38,43,54,57,59,63,71,73,74,81,97,98,99,100]
QCM-D	Change in resonance frequency (Δf) and energy dissipation factor (D)	Not applicable	Real-time, nanoscale analysis of surface phenomena (thin film formation, interactions, and reactions)	Simplifying assumptions in use of Sauerbray or other models, difficulty in interpretation	[23,25,106,107]
Nano- and micro- indentation	Hardness (H) and effective elastic modulus (E_eff_)	a/R^*^< 0.3	Surface hardness and elastic modulus	Conventional calculation of elastic modulus is limited to linear and isotropic materials	[34,61,70,116,117,122]
Peel (or adhesion) test	Adhesion strength	Depends on the peel angle (90° or 180°)	Adhesion strength between the coating and the substrate	Reliable only for tough, flexible coatings	[36,43,61,122]
Three-point bend fatigue test	Maximum stress $\left(\sigma_{max}\right)$	*L* > 4 *W*, *W* > 2 *B* ^#^	Fatigue strength versus cycles’ number	Sensitive to specimen, loading geometry, and strain rate	[61]

* *R* is the radius of the specimen and *a* is radius of the contact area between indenter and specimen surface. ^#^ *L* is the length, *B* is the width, and *W* is the height of the span.

## Abbreviations

AFM	Atomic force microscopy
AgNPs	Silver nanoparticles
ASTM	American Society for Testing and Materials
ATR-FTIR	Attenuated total reflectance Fourier-transform infrared spectroscopy
BE	Binding energy
CA	Contact angle
CaP	Calcium phosphate
CH	Chitosan
Dex	Dexamethasone
DNA	Deoxyribonucleic Acid
ECTFE	Ethylene chlorotrifluoroethylene
EPD	Electro-phoretic deposition
E-QCM	Electrochemical quartz crystal microbalance
ESCA	Electron Spectroscopy for Chemical Analysis
ESEM	Environmental scanning electron microscopy
FESEM	Field emission scanning electron microscope
GelGO	Graphene Oxide cross-linked Gelatin Fillers
GelGOHA	Graphene Oxide cross-linked Gelatin Fillers dispersed in hydroxyapatite
GL	Gelatin
Gly	Glycine
GO	Graphene oxide
HA	HydroxyApatite
HTCS	N-Hexyltrichlorosilane
Lig	Lignin
MPC	2-(Methacryloyloxy)ethyl Phosphoryl Choline
MWNT	Multi-walled nanotubes
NaPSS	Poly(Sodium-4-stryrensulfonate)
P(HEMA-MOEP)	Poly-2(Hydroxyethyl Methacrylate-2-Methacryloyloxyethyl Phosphate)
PCL	Poly(ε-Caprolactone)
PDA	Poly(Dopamine)
PEEK	Poly(Ether Ether Ketone)
PEG	Poly(Ethylene Glycol)
PEGDA	Poly(Ethylene Glycol Diacrylate)
PHEMA	Poly(2-Hydroxyethyl Methacrylate)
PLGA	Poly(D,L-Lactic-Co-Glycolic acid)
PPy	Poly(Pyrrole)
PVS	Poly(Vinylsulfonic acid, Sodium salt)
PyHTCS	6-(1,0-Pyrrolyl)-N-Hexyltrichlorosilane
Py-PD	4-Pyrrolyphenyldiazonium
QCM-D	Quartz Crystal Microbalance with Dissipation Monitoring
RGD	Arginyl-glycyl-aspartic Acid
SAMs	Self-assembled monolayers
SEM–EDX	Scanning Electron Microscopy with Energy-Dispersive X-ray analysis
SF	Silk fibroin
TEM	Transmission electron microscopy
TNs	Tio_2_-nanotubes
TNTA	Titania nanotube arrays
TOF-SIMS	Time-of-flight secondary ion mass spectrometry
XPS	X-ray photoelectron spectroscopy
